# Health System Shock Frameworks Between Theory, Application, and Assessment: Comment on "The COVID-19 System Shock Framework: Capturing Health System Innovation During the COVID-19 Pandemic"

**DOI:** 10.34172/ijhpm.2022.7373

**Published:** 2022-10-03

**Authors:** Ayman Fouda, Naomi Moy

**Affiliations:** ^1^Center for Social & Health Innovation, MCI | The Entrepreneurial School, Innsbruck, Austria.; ^2^School of Economics & Finance, Faculty of Business & Law, Queensland University of Technology, Brisbane, QLD, Australia.

**Keywords:** COVID-19, Preparedness Frameworks, Australia, Health System, Health Policy

## Abstract

The COVID-19 System Shock Framework (CSSF) tested the resilience of service providers throughout the coronavirus disease 2019 (COVID-19) pandemic in Australia. In this commentary, we tackle the topic of the CSSF applicability in less mature health systems and propose elements or dimensions that could be added to provide comprehensive response to future shocks. We acknowledge the fact that information systems, telehealth, and standard operation procedures constitute important pillars of system shock frameworks. However, there are doubts on the applicability of such pillars in middle- and low-income countries where the infrastructure is weaker compared to high-income countries and the digital divide is wider. Moreover, while it provided a paramount solution to deliver health services during the pandemic, the negative impact of telehealth should be addressed. In addition, we propose that CSSF should consider focusing on the continuity of the other medical conditions, which may have been affected due to the mitigation policies. Finally, we propose adding a dimension on the evaluation of CSSF to provide quantifiable and comparable assessment with other providers or systems.

 A new study by Hodgins et al investigated the health system response to the shock of COVID-19 using the Sydney Children’s Hospital Network as a case study.^1^ The study summarizes a specific health system response within the broader national Australian response to the pandemic and proposes the COVID-19 System Shock Framework (CSSF) to map innovations and initiatives implemented. The CSSF provides a map for the innovations to the health system that occurred throughout the first wave of the coronavirus disease 2019 (COVID-19) pandemic in Australia. The CSSF is based upon the Hanefeld et al framework, which keeps the transition of values and governance but expands upon the dimensions to include health services, health workforce, information systems, products and technologies, funding, and finance, that come together to understand what would make a ‘resilient’ health system.^2^ The original framework that the CSSF is expanded upon, was developed to examine the resilience of health systems and how they respond to shocks and studied in low- to middle-income countries in order to identify how health systems developed and responded to shocks. This framework tool aims to categorize and provide an understanding of the health procedures implemented in response to the COVID-19 pandemic. Indeed, enhancing a health system through, *inter alia*, the World Health Organization (WHO) six system building blocks,^3^ by planning early warning, and effective risk management tools, promoting primary care, with significance attached to people-centered service delivery, is paramount and cost-effective to construct a resilient health system.

 In this commentary, we address a few points to be considered for the development, extrapolation, and assessment of the CSSF. Indeed, the COVID-19 pandemic has affected different parts of the healthcare system and garnered different response strategies across and within nations. The CSSF provides interesting pillars for building blocks and assessing COVID-19 preparedness plans and resilience for health systems and service providers. However, CSSF and other system shock frameworks, to some extent, are too ideal to be extrapolated to middle- and low-income countries whose health systems are not as mature as high-income countries. This issue is especially manifested in the information systems and medical technology dimensions, where the weak infrastructure and the digital divide between countries and sub nationally have a negative impact on the extrapolation of system shock frameworks in non-high-income countries and the effectiveness of prevention and control measures.^4^

 Nevertheless, telehealth has had a monumental impact on the delivery of healthcare services in the last few decades.^5^ The dimensions of medical products and technology encompass the telehealth response used by health systems. Telehealth has been proven to offer reassurance to those suffering long-term healthcare conditions. However, the framework does not effectively demonstrate within the dimension the effect it has on patients and healthcare workers. Whilst this is demonstrated within the use of the case study – it does not incorporate negative effects and how that impacts the health systems resilience. Furthermore, the use of telehealth within the scope of CSSF can be extended not only to COVID-19 cases but also to report, monitor, and manage other patients with different medical conditions whose services might be disrupted by the mitigation policies or by the fear of visiting health facilities during the pandemic.^6^ This issue, in particular, should be reflected in future research where the application of preparedness frameworks is studied.

 In addition to telehealth, several health innovations have been developed and built into the health system response, eg, vaccination for severe acute respiratory syndrome coronavirus 2 (SARS-CoV-2), and the development of improved respiratory machines. The rollout of vaccinations would affect the health system response and influence several of the dimensions within the CSSF but are not mentioned. In the same vein, the CSSF highlights the complexities of standard operating procedures (SOPs) and how they are built in as innovations to drive behaviors in response to a pandemic and contribute to the health system resilience. In low- and middle-income countries, the extent and number of SOPs used by the Sydney Children’s Hospital Network may not be achievable, therefore, future work should extrapolate on what forms of COVID-19 related SOPs did improve health system resilience.

 Finally, the assessment of the performance of healthcare service providers during unprecedented events such as COVID-19 should also be quantified by providing statistics on the relevant health outcomes. Indeed, the inductive qualitative research methods yield important insights from the study participants. However, complementing these insights with at least the basic health indicators is paramount to provide a more comprehensive approach. Such quantification should be compared to periods prior to the shock for the same health provider and to numbers of other providers in the same period – a quasi-control group. It could also be used to assess cross-country and subnational disparities when facing health shocks.

## Ethical issues

 Not applicable.

## Competing interests

 Authors declare that they have no competing interests.

## Authors’ contributions

 AF and NM have equally contributed to the conceptualization, drafting, and replying to reviewers’ remarks.
